# Moderate hyperoxic versus near-physiological oxygen targets during and after coronary artery bypass surgery: a randomised controlled trial

**DOI:** 10.1186/s13054-016-1240-6

**Published:** 2016-03-10

**Authors:** Bob Smit, Yvo M. Smulders, Monique C. de Waard, Christa Boer, Alexander B. A. Vonk, Dennis Veerhoek, Suzanne Kamminga, Harm-Jan S. de Grooth, Juan J. García-Vallejo, Rene J. P. Musters, Armand R. J. Girbes, Heleen M. Oudemans - van Straaten, Angelique M. E. Spoelstra - de Man

**Affiliations:** Department of Intensive Care, VU University Medical Center, Amsterdam, The Netherlands; Department of Internal Medicine, VU University Medical Center, Amsterdam, The Netherlands; Department of Anaesthesiology, VU University Medical Center, Amsterdam, The Netherlands; Department of Cardiothoracic Surgery, VU University Medical Center, Amsterdam, The Netherlands; Department of Molecular Cell Biology & Immunology, VU University Medical Center, Amsterdam, The Netherlands; Department of Physiology, VU University Medical Center, Amsterdam, The Netherlands

**Keywords:** Hyperoxia, Oxygen, Intensive care unit, Cardiac surgery, CABG

## Abstract

**Background:**

The safety of perioperative hyperoxia is currently unclear. Previous studies in patients undergoing coronary artery bypass surgery suggest reduced myocardial damage when avoiding extreme perioperative hyperoxia (>400 mmHg). In this study we investigated whether an oxygenation strategy from moderate hyperoxia to a near-physiological oxygen tension reduces myocardial damage and improves haemodynamics, organ dysfunction and oxidative stress.

**Methods:**

This was a single-blind, single-centre, open-label, randomised controlled trial in patients undergoing elective coronary artery bypass surgery. Fifty patients were randomised to a partial pressure of oxygen in arterial blood (P_a_O_2_) target of 200–220 mmHg during cardiopulmonary bypass and 130–150 mmHg during intensive care unit (ICU) admission (control group) versus lower targets of 130–150 mmHg during cardiopulmonary bypass and 80–100 mmHg at the ICU (conservative group). Primary outcome was myocardial injury (CK-MB and Troponin-T) at ICU admission and 2, 6 and 12 hours thereafter.

**Results:**

Weighted P_a_O_2_ during cardiopulmonary bypass was 220 mmHg (interquartile range (IQR) 211–233) vs. 157 (151–162) in the control and conservative group, respectively (*P* < 0.0001). During ICU admission, weighted P_a_O_2_ was 107 mmHg (86–141) vs. 90 (84–98) (*P* = 0.03), respectively. Area under the curve of CK-MB was median 23.5 μg/L/h (IQR 18.4–28.1) vs. 21.5 (15.8–26.6) (*P* = 0.35) and 0.30 μg/L/h (0.25–0.44) vs. 0.39 (0.24–0.43) (*P* = 0.81) for Troponin-T. Cardiac index, systemic vascular resistance index, creatinine, lactate and F2-isoprostane levels were not different between groups.

**Conclusions:**

Compared to moderate hyperoxia, a near-physiological oxygen strategy does not reduce myocardial damage in patients undergoing coronary artery bypass surgery. Conservative oxygen administration was not associated with increased lactate levels or hypoxic events.

**Trial registration:**

Netherlands Trial Registry NTR4375, registered on 30 January 2014

**Electronic supplementary material:**

The online version of this article (doi:10.1186/s13054-016-1240-6) contains supplementary material, which is available to authorized users.

## Background

During cardiac surgery, and especially during cardiopulmonary bypass (CPB), patients often receive supplemental oxygen as a precaution to prevent hypoxia. As a result, the arterial oxygen tension (partial pressure of oxygen in arterial blood; P_a_O_2_) often rises to supraphysiological levels (hyperoxia), which is by many considered as a salutary oxygen reserve. P_a_O_2_ levels of more than 200 to 300 mmHg during CPB are not exceptional [1–6] and hyperoxia is also common in mechanically ventilated patients in the intensive care unit (ICU) [7, 8]. There are currently no guidelines on oxygenation during cardiac surgery. For intensive care, current target P_a_O_2_ recommendations are limited to patients with acute respiratory distress syndrome [9].

The safety of hyperoxia is questionable [10, 11]. Besides the direct toxic effect of high fractions of inspired oxygen on lung tissue, animal [12–14] and human studies [15–19] show that hyperoxia induces vasoconstriction and may reduce cardiac output and organ perfusion. The mechanism of hyperoxic vasoconstriction is unclear, but augmented production of reactive oxygen species (ROS) seems to play a pivotal role [20, 21]. Excessive ROS may, amongst other things, enhance ischaemia/reperfusion injury, which may translate into worse clinical outcome.

In patients after cardiac arrest, hyperoxia is associated with worse functional outcome and increased mortality [22–26]. In patients with cerebral [27] or myocardial infarction [28], hyperoxia has been shown to increase infarct size and mortality. Data on the effect of oxygen on cardiac markers are scarce, since in animal studies direct measurements of infarct size are preferred and human studies are lacking. However, the recently published AVOID trial shows increased Troponin-I and creatine kinase (CK) when patients received supplemental oxygen after myocardial infarction [28].

Patients undergoing coronary artery bypass graft (CABG) surgery may be particularly vulnerable to the effects of hyperoxia because of fluctuations in cardiac function due to anaesthesia, blood loss, altered temperature, non-pulsatile perfusion and fluid shifts post-surgery. Also, hyperoxia may have detrimental effects on CPB-related myocardial ischaemia/reperfusion injury, pulmonary reperfusion and systemic inflammation.

The few clinical trials addressing the effect of hyperoxia during or after CABG surgery [1–6, 29] suggest that avoidance of extreme hyperoxia reduces myocardial damage (>400 mmHg) [1, 2]. The design of these studies is variable, with exposure of patients to single [4, 5] or multiple short episodes [6] of hyperoxia and application of different oxygen tensions during reperfusion [2] or continuously throughout CPB [1]. In recognition of direct pulmonary toxic effects of high inspiratory oxygen fractions these extreme P_a_O_2_ values (~400 mmHg) are no longer used in current practice. Nevertheless, currently employed oxygen levels remain high, which raises the question whether further P_a_O_2_ reductions are beneficial. Additionally, no studies continued to control the arterial oxygen tension after admission to the ICU.

The aim of this randomised controlled trial (RCT) was to determine whether near-physiological oxygen targets during and after CABG surgery reduces myocardial damage and improves haemodynamics, organ function and oxidative stress compared to standard care, which involves moderate hyperoxia.

## Methods

### Study design and ethical approval

This investigator-initiated, single-centre, RCT (Netherlands Trial Register number NTR4375) was performed at the operating room (OR) and ICU of the VU University Medical Centre (Amsterdam, the Netherlands). The study protocol was approved by the Dutch Central Committee on Research Involving Human Subjects (NL43882.029.13).

### Patients

Patients over 18 years of age with a body surface index ≥1.9 m^2^ and a preoperative haemoglobin concentration ≥121 g/L, scheduled for elective, isolated CABG surgery with CPB were approached for participation and included in the study after written informed consent. Body surface index and haemoglobin criteria were chosen to ensure sufficient oxygen-carrying capacity of blood after haemodiluton by the CPB circuit. Exclusion criteria were presence of a perioperative intra-aortic balloon pump or a medical history positive for chronic obstructive pulmonary disease.

### Clinical management

#### Anaesthesia

Anaesthesia was induced with a combination of sufentanil (3–7 μg/kg), midazolam (0.1 mg/kg) and pancuroniumbromide (0.1 mg/kg) or rocuronium (0.6 mg/kg). General anaesthesia was maintained using propofol (200–400 mg/h). After induction, all patients received dexamethasone (1 mg/kg), cefazoline (1000 mg) and tranexamic acid (1000 mg). Another 2000 mg of tranexamic acid was administered following termination of CPB. After surgery, patients were supported with a low dose of dopamine.

#### Cardiopulmonary bypass

Non-pulsatile CPB was initiated in a standard fashion with moderate hypothermia (34–36 °C). Depending on the operating surgeon, cardiac arrest was achieved by using 4 °C crystalloid cardioplegia solution or normothermic blood cardioplegia. After surgery, shed blood was washed, concentrated and returned to the patient. For more details, see Additional file 1.

#### Intensive care unit

Propofol sedation was stopped when haemodynamics were stable, chest drain production was minimal and patient temperature was normal. When sufficiently awake, patients were extubated. Patients received three gifts of cefazoline (1000 mg) at 8-h intervals.

### Randomisation and intervention

After inclusion, patients were randomised by drawing of sealed envelopes into the “control” or “conservative” group (1:1 ratio). Patients were enrolled and assigned by BS, who was not involved in the clinical care of patients. While on CPB, the control group was oxygenated, aiming for a target P_a_O_2_ of 200–220 mmHg during placement of the aortic cross-clamp as per current practice. After admission to the ICU, a target P_a_O_2_ of 130–150 mmHg was applied. The conservative group was oxygenated to a target P_a_O_2_ of 130–150 mmHg during aortic cross-clamping and 80–100 mmHg at the ICU. Surgeons were unaware of group allocation.

Patients in both study groups were pre-oxygenated with a fraction of inspired oxygen (F_I_O_2_) of 1.0 and subsequently intubated. In the control group, F_I_O_2_ was set to a minimum of 0.4 and a maximum of 0.6. In the conservative group, F_I_O_2_ was set lower than or equal to 0.40, while ensuring an oxygen saturation ≥94 %. During CPB, P_a_O_2_ was continuously monitored with a Terumo CDI 500 (Terumo Europe, Leuven, Belgium). After ICU admission, P_a_O_2_ was monitored approximately every hour by blood gas sampling. While patients were intubated, F_I_O_2_ was adjusted (with an upper limit of 60 %, higher only if clinically necessary) to reach target P_a_O_2_. After extubation, patients breathed room air or received supplemental oxygen through a nasal cannula or an oxygen mask. Other means of non-mechanical ventilation were only issued on clinical indication and not for study purposes.

### Primary and secondary endpoints

The primary endpoint was myocardial damage, as reflected by area under the curve (AUC) of CK-MB. Secondary endpoints were differences in haemodynamics, tissue perfusion, organ function, and oxidative stress. As safety outcomes, we monitored for desaturations (oxygen saturation <88 %) and hypoxic events (P_a_O_2_ < 55 mmHg).

#### Myocardial damage/tissue perfusion, organ function and injury

Myocardial damage was determined by measuring CK-MB, Troponin-T and CK at the following time points: prior to induction of anaesthesia and 0, 2, 6 and 12 h after ICU admission. If no peak value was measured within this timeframe, additional measurements were performed at 6-h intervals. As an estimation of organ perfusion and oxygenation, creatinine and lactate levels were measured at the same time points. Lung injury score (LIS) was assessed at ICU admission and 2 days post-operatively [30]. P_a_O_2_/F_I_O_2_ ratio was measured approximately every hour, simultaneously with blood gas analyses for intervention control.

#### Haemodynamics

In patients who received a pulmonary artery catheter (under the anaesthesiologist’s discretion), cardiac index (CI) and systemic vascular resistance index (SVRI) were measured prior to thoracotomy, after chest closure and 0, 2, 6 and 12 h after ICU admission.

#### Oxidative stress

Lipid peroxidation by ROS was assessed by measuring F2-isoprostanes (8-iso prostaglandin F2α, iPF2α-III) in plasma by liquid chromatography tandem mass spectrometry. EDTA samples were obtained prior to induction of anaesthesia and 6 h after ICU admission. Directly after sampling, blood was centrifuged at 2500 g for 10 minutes and plasma was stored at –80 °C until analysis.

For the measurement of ROS production by circulating polymorphonuclear leukocytes (PMN), samples were taken before induction of anaesthesia and at 1 h of CPB. A portion of whole blood was incubated with a fluorescent ROS probe for 1 h (CellROX Green; Invitrogen, Grand Island, NY, USA). Mean fluorescence intensity (MFI) of 5000 cells, which is proportionate to the ROS produced, was then measured on a Beckton Dickinson FACS Calibur flowcytometer.

For a more elaborate description of these methods, see Additional file 2.

### Statistics/analysis

#### Sample size calculation

A previous small trial, which compared extreme versus less extreme hyperoxia during CPB, showed a perioperative increase in CK-MB of 20 to 80 μg/L and 20 to 45 μg/L, respectively [2]. In the present trial, we expected the effect to be smaller. With an anticipated standard deviation of 20 μg/L and an α of 0.05, inclusion of 25 patients in each arm provides a power of 80 % to detect a CK-MB difference of 20 %.

#### Analysis

Data were analysed according to the intention-to-treat principle (ITT). Data are presented as median and interquartile range (IQR) unless otherwise stated. AUC was calculated using the Trapezoidal Rule. Time-weighted P_a_O_2_ was calculated by multiplying the last P_a_O_2_ of two consecutive measurements by the period of time between these measurements; the sum of all products was then divided by total time. Weighted P_a_O_2_/F_I_O_2_ ratios were calculated following the same principle. Differences between groups were tested with the non-parametric Mann-Whitney U test or Fisher’s exact test where appropriate.

## Results

### Patients

In the period between November 2013 and May 2015, 57 patients gave written informed consent for study participation. Seven patients were excluded prior to the start of the study protocol due to changes in the surgery schedule. The remaining 50 patients, 25 in each arm, completed the study protocol and were included for ITT analysis. The participants were Caucasian males with a median age of 67 years (IQR 63–71). For more detailed baseline and surgical characteristics of patients, see Table 1. For a CONSORT flow diagram, see Additional file 3.

**Table 1:** Baseline and surgical characteristics

	Hyperoxia n = 25	Normoxia n = 25
Demographics		
Age, years	66 (61–71)	68 (66–71)
Male, n (%)	25 (100)	25 (100)
Body surface area, m^2^	2.1 (1.9–2.1)	2.1 (1.9–2.0)
Caucasian race, n (%)	25 (100)	25 (100)
Medical history		
Smoker, n (%)	16 (64)	17 (68)
Diabetes, n (%)	6 (24)	5 (20)
Hypertension, n (%)	7 (28)	13 (52)
Hypercholesterolaemia, n (%)	5 (25)	8 (32)
Renal insufficiency, n (%)	1 (4)	5 (20)
Cardiac arrest, n (%)	1 (4)	1 (4)
Cerebrovascular accident, n (%)	1 (4)	2 (8)
Cardiovascular history		
Atrial fibrillation, n (%)	3 (12)	2 (8)
Myocardial infarction, n (%)	14 (56)	8 (32)
PCI, n (%)	6 (24)	6 (24)
Left ventricular function		
Good, n (%)	19 (76)	17 (68)
Moderate, n (%)	5 (20)	6 (24)
Poor, n (%)	1 (4)	2 (8)
Baseline laboratory parameters		
Troponin-T (μg/L)	0.012 (0.009–0.016)	0.014 (0.009–0.031)
CK (U/L)	86 (57–126)	77 (56–102)
CK-MB (μg/L)	0 (0–1.9)	0 (0–0)
Serum creatinine (mmol/L)	78 (69–93)	88 (74–108)
Haemoglobin (g/L)	142 (139–148)	140 (132–148)
Surgical characteristics		
Number of grafts, n	4 (3–4)	4 (4–5)
Surgery time, min	240 (212–283)	245 (220–280)
CPB time, min	103 (85-122)	103 (95-133)
Cross clamp time, min	66 (56-80)	69 (60-85)
Blood cardioplegia, n (%)	2 (8)	6 (24)

Data are presented as median (interquartile range) unless otherwise stated. Renal insufficiency was defined as an estimated glomerular filtration rate <60 mL/min. *CK* creatine kinase, *CPB* cardiopulmonary bypass, *MB* muscle/brain, *PCI* percutaneous coronary intervention

### Intervention

In the control group, weighted P_a_O_2_ during CPB was 220 mmHg (213–233) versus 157 (152–161) (*P* < 0.0001) in the conservative group. The lowest haemoglobin concentration during CPB was 92 g/L (87–97) vs. 89 (84–95) (*P* = 0.170). During aortic cross-clamping, weighted P_a_O_2_ was 214 mmHg (207–224) vs. 146 (141–151) (*P* < 0.0001). During ICU admission, weighted P_a_O_2_ was 107 mmHg (88–140) vs. 90 (85–97) (*P* = 0.034), respectively. Haemoglobin concentrations were 119 g/L (111–126) vs. 119 (113–122) (*P* = 0.690) on ICU admission. Patients in the control group were on mechanical ventilation for 4.1 h (3.2–5.0) with an F_I_O_2_ of 0.52 (0.43–0.60). Patients in the conservative group were on mechanical ventilation for 4.3 h (3.1–6.2) (*P* = 0.383) with an F_I_O_2_ of 0.38 (0.32–0.44) (*P* < 0.0001).

### Post-operative course

Three patients underwent a re-thoracotomy for post-operative bleeding (one in the control group vs. two in the conservative group). One patient in the conservative group then developed cardiogenic shock (lowest P_a_O_2_ = 76 mmHg). Two patients (one in either group) were scheduled for percutaneous coronary intervention after full recovery due to incomplete revascularisation. One patient in the conservative group had a ST-segment elevation myocardial infarction (STEMI) during ICU admission that did not require additional treatment (CK-MB_max_ = 74.9 μg/L). None of these events led to prolonged ICU admission. Patients in the control group were admitted to the ICU for 22 h (19–24) versus 22.5 h (19.5–24.5) for patients in the conservative group (*P* = 1.000).

### Myocardial damage

**Fig. 1:**
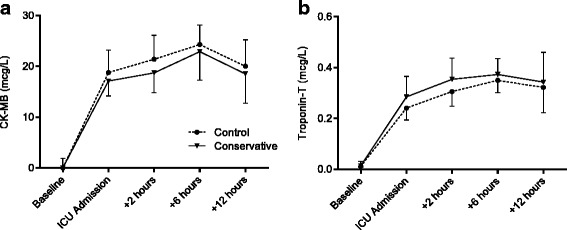
Perioperative **a** CK-MB and **b** Troponin-T levels. There were no differences between groups. *CK-MB* creatine kinase—muscle/brain, *ICU* intensive care unit

CK-MB values generally peaked at 6 h after ICU admission (Fig. 1a). AUC were 23.5 μg/L/h (18.4–28.1) in the control group vs. 21.5 (15.8–26.6) (*P* = 0.347) in the conservative group. Individual maximum values were 25.8 μg/L (21.0–32.3) vs. 24.9 (18.4–31.5) (*P* = 0.528).

Troponin-T levels rose substantially after surgery and usually peaked at 6 h after ICU admission (Fig. 1b). AUC were not different between groups (0.30 μg/L/h (0.25–0.44) vs. 0.39 (0.24–0.43); *P* = 0.695)). Individual peak values were 0.35 μg/L (0.30–0.46) vs. 0.42 (0.26–0.49) (*P* = 0.923).

CK levels rose gradually during ICU stay and did not decline in the following 12-h study period. AUC were similar at 550 U/L/h (347–706) vs. 376 (229–425) (*P* = 0.144). Individual maximum values measured were 663 U/L (403–977) vs. 511 (329–637) (*P* = 0.165).

To correct for an imbalance between groups in the number of patients who received warm blood cardioplegia (Table 1; *P* = 0.247), we performed a multiple linear regression with CK-MB AUC as the dependent variable, and study group, minutes on CPB and type of cardioplegia as independent variables. Neither study group (β = 0.024, *P* = 0.874), nor minutes on CPB (β = 0.200, *P* = 0.178) nor type of cardioplegia (β = –0.003, *P* = 0.984) were associated with CK-MB AUC (R^2^ = 0.041).

### Tissue perfusion, organ function and injury

**Fig. 2:**
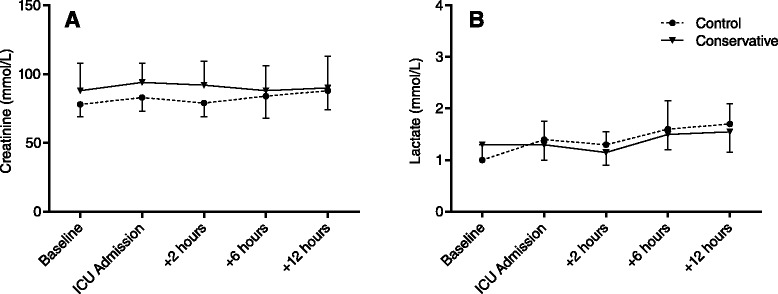
Perioperative **a** creatinine and **b** lactate levels. There were no differences between groups. *ICU* intensive care unit

Creatinine remained stable throughout the study period (Fig. 2a). Lactate levels increased mildly in both groups and were not different between groups (Fig. 2b). On ICU admission, lung injury scores were 1.3 (0.8–1.5) in the control group vs. 1.0 (0.7–1.5) (*P* = 0.290) in the conservative group. Two days later, LIS was 1.0 (0.8–1.5) vs. 1.0 (0–1.5) (*P* = 0.216). During ICU admission, weighted P_a_O_2_/F_I_O_2_ ratios were 236 (173–285) vs. 261 (199–311) (*P* = 0.244).

### Haemodynamics

In 15 patients in the control group a pulmonary artery catheter was inserted. Preoperatively, 11 had good Left Ventricular Function (LVF), 3 moderate and 1 poor. In the conservative group, 18 patients received a pulmonary artery catheter, with 10 having good LVF, 6 moderate and 2 poor.

**Fig. 3:**
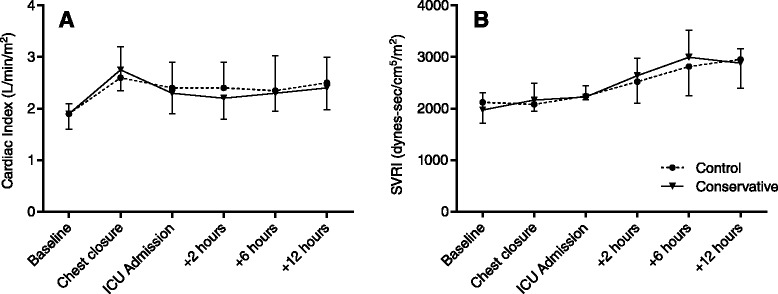
Perioperative haemodynamic parameters. **a** Cardiac index and **b** SVRI. There were no differences between groups. *ICU* intensive care unit, *SVRI* systemic vascular resistance index

Before sternotomy, CI was similar between groups (1.9 L/min/m^2^ (1.6–2.1) vs. 1.9 (1.6–2.2); *P* = 1.000). After chest closure, CI was increased compared to baseline in both groups (2.6 (2.2–3.2) vs. 2.8 (2.4–3.0)). After admission to the ICU, CI decreased slightly and then remained stable throughout the next 12 hours (Fig. 3a). There was no difference between the two groups at any point. SVRI rose gradually in both groups (Fig. 3b), starting at 2120 dynes · s/cm^5^/m^2^ (1711–2254) in the control group and 1970 (1787–2311) in the conservative group and reaching a maximum at 12 h of ICU admission (2955 (2398–3351) vs. 2883 (2564–3161); *P* = 0.861).

Nearly all patients in either group were supported with a low dose of dopamine in the first hours after ICU admission (22/25 in the control group vs. 20/25 in the conservative group). Overall administration of vasoactive medicine was not different between groups (data not shown).

### Oxidative stress

At baseline, there was no difference in F2-isoprostane levels between groups (32.1 ng/L (26.7–36.0) in the control group vs. 34.3 (24.8–43.2) in the conservative group; *P* = 0.467). Six hours after ICU admission, F2-isoprostane levels were lower in both groups compared to baseline (*P* < 0.001), but there was no difference between groups (25.9 ng/L (23.7–28.5) vs. 27.3 (22.6–31.6); *P* = 0.399).

ROS production by PMN increased in the control group from a mean 21.3 MFI (95 % confidence interval 14.6–28) at baseline to 45.9 (21.8–70.0) (*P* = 0.001) at 1 h of CPB. In the conservative group, ROS production increased similarly from 15.6 MFI (10.1–20.7) at baseline to 45.2 (14.5–76.0) (*P* = 0.002) during CPB. There was no difference between groups either at baseline (*P* = 0.228) or at 1 h of CPB (*P* = 0.790).

### Safety outcome

All patients that participated in the study survived until hospital discharge. There were no hypoxic events (P_a_O_2_ < 55 mmHg) in either study group.

## Discussion

In the present RCT in patients undergoing scheduled isolated CABG surgery, we found no difference in markers of myocardial damage between a conventional moderate hyperoxic and a near-physiological oxygen strategy during and after surgery. The conservative oxygenation strategy did not improve haemodynamics in comparison to moderate hyperoxia. However, lower oxygen targets during CPB and ICU admission could be applied safely without risk of hypoxia.

With respect to myocardial damage, these findings are in apparent contrast to results from earlier clinical trials where the reduction of oxygen tension during CPB or reperfusion resulted in lower CK (672 ± 130 vs. 293 ± 21 U/l; *P* = 0.002) [1] or lower Troponin-T levels (~2.1 vs. ~0.8 μg/L; *P* < 0.05) [2]. However, the high P_a_O_2_ investigated in the latter two studies are no longer applied today. The first study compared a P_a_O_2_ of ~400 mmHg during CPB in the control group to ~140 mmHg in the conservative group [2]. The second study compared reperfusion oxygen tensions of 450–550 mmHg to 200–250 mmHg [1]. This means that, in the conservative group, oxygen tension was reduced by 260–270 mmHg compared to the control group. In our study, the difference was on average 70 mmHg during CPB and 20 mmHg during ICU admission. It is reasonable to assume that a smaller reduction in oxygen tension has a smaller effect on myocardial damage.

Our results are in contrast to those of the AVOID trial, which compared the effect of 8 L/min oxygen vs. no-oxygen supplementation in non-hypoxic patients with STEMI. The study showed increased CK, a trend for increased Troponin-I and increased infarction size when patients received supplemental oxygen [28]. Unfortunately, the study was oxygen saturation-guided and there are no P_a_O_2_ values available, so we cannot deduce whether oxygen tensions were in the same range. Of course, an obvious difference between the patient populations is that, in STEMI, the insult is acute, while in CABG surgery the patient is prepared with moderate hypothermia and cardioplegia. This fundamental physiological difference may explain the contrasting results.

In addition, an important difference with previous CABG studies is that our patients received 1 mg/kg dexamethasone after anaesthesia induction [31]. Dexamethasone is a potent glucocorticoid that reduces the formation of ROS by leukocytes in vivo [32] and, in CABG surgery, reduces the systemic inflammatory response syndrome [33, 34]. Since it is hypothesised that hyperoxia exerts its effects by augmenting production of ROS, it is possible that dexamethasone mitigates these effects. In anaesthetised [26, 33] and septic patients [34] supplemental oxygen can decrease CI and increase SVRI [14]. In these studies the contrast between the normoxic and hyperoxic state was high (i.e. switching from an F_I_O_2_ of ~0.3 to 1.0) and short term (10–30 min). In our study, moderate hyperoxia appears to have no effect on CI or SVRI. Although the relatively lower P_a_O_2_ difference between groups post-operatively may have led to a minor and immeasurable difference in haemodynamic parameters, it is also possible that the haemodynamic effects are only temporary. Also, dexamethasone influences vascular endothelial and smooth muscle Ca^2+^ mobilisation [35], which in turn modulates the balance between vasoconstriction and vasodilation which may have affected the vascular response to oxygen.

Over the years, CABG surgery has improved and the influence of oxygen tension may be less in current high-quality surgery and post-operative care. This view is supported by observation that cardiac markers in the present study appear lower than in earlier studies. For instance, the lowest maximal Troponin-T reported in one study was ~0.8 ng/mL and the lowest maximal CK-MB ~50 ng/mL [2]. In our study, we found levels that were approximately 50 % lower (0.42 ng/mL for Troponin-T and 24.9 ng/mL for CK-MB). It should be noted, however, that the actual difference may be slightly different, since the assays to detect these markers have changed as well. However, in the case of Troponin-T, all commercial assays have been using the same set of antibodies for years and comparability between assay generations is good in the upper range [36, 37].

The present study shows that employing a strategy with lower oxygenation targets can be performed safely in low-risk CABG surgery. There were no hypoxic events and there was no increase in lactate or creatinine levels, indicating adequate aerobic metabolism. This finding is in agreement with another study that focussed solely on strategies to avoid hyperoxia during CPB [3]. Only one other (retrospective) study reported post-operative creatinine levels, and, like our results, they did not find a difference between the control and conservative group [38].

### Study limitations

Although we anticipated that the current intervention would possibly find a smaller effect on cardiac markers compared to earlier trials [1, 2], we did not foresee the overall lower levels of Troponin-T and CK-MB. A revised power calculation with the present numbers indicates that 51 patients in either arm would be needed to detect a CK-MB difference of 20 % with 80 % power. However, amending the protocol to increase the sample size was deemed futile. If the sample size was to be doubled, the conditional power to detect the hypothesised effect (20 % difference in CK-MB) was 46 %. Assuming that the current trend continued, the conditional power would be only 11 %.

The P_a_O_2_ of some patients in the control group were lower than the desired targets. Due to the toxicity of oxygen for lung tissue, we found it unethical and not compatible with daily practice to increase F_I_O_2_ above 0.60 for study purposes only. In these cases, we accepted adequate, but lower than target, P_a_O_2_. Although we think that it may have reduced differences between groups, it is unlikely that it entirely explains our results. For one, in previous studies P_a_O_2_ was controlled during surgery only, suggesting that in CABG surgery intraoperative oxygenation has the more profound impact on myocardial damage [1, 2]. Secondly, weighted P_a_O_2_ was different between groups during ICU admission and therefore differential oxygen exposure was longer in our trial than in previous ones.

Despite randomisation, slightly more patients in the conservative group received warm blood cardioplegia as opposed to crystalloid cardioplegia. We do not think that this small imbalance had severe effects on the results of this trial. Although the literature is far from conclusive, warm blood cardioplegia appears to be related to a small reduction of CK-MB release after surgery [39]. At best, this could have led to a false positive—that a reduction of oxygen tension reduces cardiac damage. In addition, we did not find an association between type of cardioplegia and CK-MB AUC using multiple linear regression.

All patients endured at least three short episodes of hyperoxia: 1) during pre-oxygenation; 2) during simultaneous oxygenation through the endotracheal tube and the extracorporeal oxygenator; and 3) during transport from the OR to the ICU. That these short periods of hyperoxia explain our results seems unlikely, but cannot be ruled out either.

The current study was performed in a low-risk CABG population. The same intervention in high-risk patients or during combined surgery (CABG + valvular surgery) may give different results.

To reduce the impact of CPB haemodilution on oxygenation, we included patients with a minimum body size and pre-operative haemoglobin level. Patients that are more affected by the volume of the CPB circuit (i.e. smaller or anaemic patients) might respond differently to either of the two oxygen strategies. Additionally, due to these criteria (especially body surface index) nearly all eligible participants were male, which makes the results of this trial gender specific.

### Clinical implications and future directions

The most important note from this trial for clinical perfusionists and physicians is that lower arterial oxygen targets can be applied safely during CPB and post-operative intensive care. These results are in accordance with several recently published implementation studies for conservative oxygen therapy in the ICU [40–42].

With regard to the effects of hyperoxia on the cardiovascular system, it seems that perfusionists and physicians do not have to be particularly concerned about possible oxygen-induced haemodynamic disturbances in uncomplicated post-CABG patients, at least in the range of 80–150 mmHg.

Now that it is becoming clearer that aiming for lower arterial oxygen tensions is not harmful, we feel it is time for much larger RCTs in order to find optimal oxygenation targets in high-risk and combined cardiac surgery and intensive care. With this thought, we have initiated a multicentre RCT (O2-ICU, NCT02321072) to investigate optimal oxygenation targets in the ICU.

## Conclusion

In the present RCT in elective isolated CABG surgery and post-operative intensive care, an oxygenation strategy towards near-physiological arterial oxygen tensions did not result in less myocardial damage or improved haemodynamics in comparison to moderate hyperoxia. However, the conservative oxygen strategy was safe and can be employed without concern for inadequate aerobic metabolism. Future, much larger RCTs are required to determine optimal oxygenation targets in high-risk and combined cardiac surgery and intensive care.

## Key messages

- A near-physiological P_a_O_2_ oxygenation strategy during and after CABG surgery did not result in less myocardial damage, compared to moderate hyperoxia
- In elective isolated CABG surgery, a conservative oxygen strategy does not improve haemodynamics, compared to moderate hyperoxia
- A conservative oxygen strategy can be applied safely both during CPB and ICU admission without hypoxic events

## Additional files

Additional file 1: Cardiopulmonary bypass details. A more detailed description of the cardiopulmonary bypass used during the study. (DOCX 12 kb) Additional file 2: Oxidative stress method details. A more detailed description of the methods used to measure F2-isoprostanes and reactive oxygen species production by polymorphonuclear cells during the study. (DOCX 14 kb) Additional file 3: CONSORT 2010 flow diagram. Diagram of the inclusion and exclusion of study subjects according to the CONSORT 2010 statement. (DOC 47 kb)

## Abbreviations

AUC: Area under the curve
CABG: Coronary artery bypass graft
CI: Cardiac index
CK: Creatinine kinase
CPB: Cardiopulmonary bypass
F_I_O_2_: Fraction of inspired oxygen
ICU: Intensive care unit
IQR: Interquartile range
ITT: Intention-to-treat
LIS: Lung injury score
LVF: Left ventricular function
MB: Creatinine kinase - muscle/brain
MFI: Mean fluorescence intensity
OR: Operating room
P_a_O_2_: Partial pressure of oxygen in arterial blood
PMN: Polymorphonuclear leukocytes
RCT: Randomised controlled trial
ROS: Reactive oxygen species
STEMI: ST-segment elevation myocardial infarction
SVRI: Systemic vascular resistance index
