# Supplementary material for: Moderate hyperoxic versus near-physiological oxygen targets during and after coronary artery bypass surgery: a randomised controlled trial
Source: Crit Care. 2016 Mar 10;20:55. doi: 10.1186/s13054-016-1240-6 (PMC4788916; doi:10.1186/s13054-016-1240-6)
Supplement: Additional file 1: — Cardiopulmonary bypass details. A more detailed description of the cardiopulmonary bypass used during the study. (DOCX 12 kb) [file 13054_2016_1240_MOESM1_ESM.docx]

**Cardiopulmonary Bypass details**

The extracorporeal circulation system consisted of a C5 heart lung machine (Sorin/Stöckert Instrumente GmBH, Munich, Germany) with a biocompatible coated (phosphorylcholine, p.h.i.s.i.o) centrifugal pump and extracorporeal circuit with an Inspire 8FM hollow fiber oxygenator (Sorin). The circuit was primed with 750 ml gelatine solution (Gelofusin, Braun Melsungen AG, Melsungen, Germany), 500 ml lactated Ringer’s solution (Baxter BV, Utrecht, Netherlands), 100 ml 20% mannitol, 50 ml 8.4% sodium bicarbonate (Braun Melsungen AG) and 5000 IU porcine heparin (LEO Pharma, Amsterdam, The Netherlands). After heparinization, non-pulsatile cardiopulmonary bypass was initiated using an aortic arterial cannula (24 french), and a two-stage venous cannula in the right atrium (36 French). Myocardial protection was achieved by using 4°C crystalloid cardioplegia solution or normothermic blood cardioplegia, depending on the preference of the operating surgeon. Patient temperature was maintained between 34°C and 36°C and blood flow was kept between 2.4-2.6 l/min/m2. Patients were weaned from CPB when the rectal temperature reached 36°C. During CPB, mean arterial pressure was kept between 60 and 70 mmHg. After CPB, shed blood during surgery as well as the remaining volume in the extracorporeal circuit was washed and concentrated by means of a cell saver (Autolog, Medtronic, Minneapolis, MN, USA) and returned to the patient.
