# Supplementary material for: Moderate hyperoxic versus near-physiological oxygen targets during and after coronary artery bypass surgery: a randomised controlled trial
Source: Crit Care. 2016 Mar 10;20:55. doi: 10.1186/s13054-016-1240-6 (PMC4788916; doi:10.1186/s13054-016-1240-6)
Supplement: Additional file 2: — Oxidative stress method details. A more detailed description of the methods used to measure F2-isoprostanes and reactive oxygen species production by polymorphonuclear cells during the study. (DOCX 14 kb) [file 13054_2016_1240_MOESM2_ESM.docx]

Oxidative Stress Method details

**F2 Isoprostanes**

The total, i.e. free and esterified, concentration of 8-iso prostaglandin F2α (iPF2α-III) was determined by liquid chromatography tandem mass spectrometry (LC-MS/MS). In brief, 0.02 mL of 10 ng/mL deuterated internal standard (8iPF2α-d4; Cayman chemical, USA) was added to 0.4 mL EDTA-plasma. To prevent arachidonic acid from auto-oxidation during sample preparation, butylated hydroxytoluene was added to a final concentration of 3 mmol/L. Then 0.05 mL of 10 mol/L KOH and 0.4 ml MeOH were added for alkaline hydrolysis, and the samples were incubated in a shaking water bath for 60 min at 40 °C. Afterwards, the samples were cleaned up using polymeric strong anion exchange 96-well solid phase extraction (SPE) plates (Strata-X-A-33u 60 mg/well, Phenomenex, Torrance, USA). The wells were successively washed with 1 mL of 2% NH4OH, 1 mL hexane and 1 mL 2-propanol using a positive pressure-96 processor (Waters, Milford, USA). The fraction containing F2-isoprostanes was eluted with 1 mL of 0.5% acetic acid in 2-propanol and then dried under a stream of nitrogen at room temperature and redissolved in 0.1 mL 25% acetonitrile containing 0.05% acetic acid. A volume of 15 μL was injected on an Acquity BEH C18 column (Waters; 1.7 μm, 2.1 × 100 mm). F2-isoprostanes were quantified by an API 5000 triple quadrupole mass spectrometer (AB Sciex Technologies, Toronta, Canada) in negative ion multiple reaction monitoring acquisition mode. To calculate the iPF2α-III concentration, the analyte to internal standard peak area ratio with transitions 353.2 to 193.2 and 357.2 to 197.2 respectively, were compared with a standard curve up to 5.6 nmol/L iPF2α-III (Cayman chemical). The intra-run and inter-run assay variations were 4.6% and 8.2%, respectively.

**ROS production by PMN**

Samples for the measurement of ROS production by circulating Polymorphonuclear Leukocytes (PMN) were taken before induction of anaesthesia and at one hour of CPB. 30 µL of citrated whole blood was incubated with 10 µM of CellROX Green (Invitrogen, Grand Island, NY, USA) for one hour. After oxidation, the CellROX probe binds to DNA and becomes excitable. After incubation, leukocytes were isolated through lysis of erythrocytes by hypotonic shock and were washed twice with phosphate buffered saline (PBS). Positive and negative controls were obtained by parallel incubation with phorbol-12-myristate-13-acetate (Sigma Aldrich, Zwijndrecht, The Netherlands) and N-acetyl-cysteine. To compensate for the vast neutrophil count increase during CPB, the one hour CPB sample was incubated with 10, 20 and 30 µM CellROX to construct a concentration-response curve and the ROS production was calculated based on the CellROX:Neutrophil ratio in the baseline sample. PMN were counted in a separate lyse-no wash sample using counting beads (CountBright Absolute Counting Beads, Invitrogen). After the last washing step, cells were suspended in PBS and stored on ice in the dark until analysis on a Beckton Dickinson FACSCalibur flowcytometer. The fluorescent properties of PMN were measured in the FL1 channel (530/30 nm emission filter) after excitation with a 488 nm argon laser. PMN were gated based on forward- and sideward scatter characteristics to avoid activation of PMN by fluorescent cell-specific antibodies. Results are expressed as MFI of 5000 cells.
