# Supplementary material for: Moderate hyperoxic versus near-physiological oxygen targets during and after coronary artery bypass surgery: a randomised controlled trial
Source: Crit Care. 2016 Mar 10;20:55. doi: 10.1186/s13054-016-1240-6 (PMC4788916; doi:10.1186/s13054-016-1240-6)
Supplement: Additional file 3: — CONSORT 2010 flow diagram. Diagram of the inclusion and exclusion of study subjects according to the CONSORT 2010 statement. (DOC 47 kb) [file 13054_2016_1240_MOESM3_ESM.doc]

**
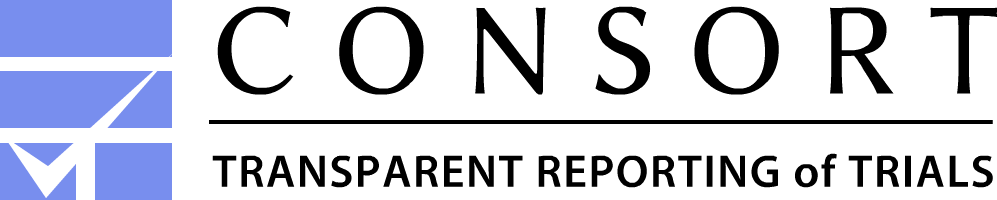
**

**CONSORT 2010 Flow Diagram**

**Allocation**

**Analysis**

**Enrollment**

Assessed for eligibility (n=404)

Excluded (n=347)

♦  Not meeting inclusion criteria (n=272)

♦  Declined to participate (n=11)

♦  Participation in conflicting study (n=43)

♦  Other reason (n=21)

Analysed (n=25)
♦ Excluded from analysis (n=0)

**Control group**

Allocated to intervention (n=28)

♦ Received allocated intervention (n=25)

♦ Did not receive allocated intervention due to change in operation schedule (n=3)

**Conservative group**

Allocated to intervention (n=29)

♦ Received allocated intervention (n=25)

♦ Did not receive allocated intervention due to change in operation schedule (n=4)

Analysed (n=25)
♦ Excluded from analysis (n=0)

Randomized (n=57)
